# 2283. COVID-19 among Febrile Patients Presenting without Respiratory Symptoms in Six Hospitals along the Thai-Lao and Thai-Myanmar Borders, 2021-2011

**DOI:** 10.1093/ofid/ofad500.1905

**Published:** 2023-11-27

**Authors:** Saithip Bhengsri, Emily Bloss, Beth Skaggs, Pongpun Sawatwong, Nuttagarn Chuenchom, Tanaphat Lertwitayakumjorn, Barameht Piralam, Samkan Chaoprasert, Tuwan Simmali, Pornpan Bungkanjana, Sutthinee Boonlong, James D Heffelfinger, Anupong Sujariyakul

**Affiliations:** Division of Global Health Protection, Thailand MoPH-US CDC Collaboration, Ministry of Public Health, Nonthaburi, Thailand, Muang, Nonthaburi, Thailand; Division of Global Health Protection, Thailand MoPH-US CDC Collaboration, Ministry of Public Health, Nonthaburi, Thailand, Muang, Nonthaburi, Thailand; Division of Global Health Protection, Thailand MoPH-US CDC Collaboration, Ministry of Public Health, Nonthaburi, Thailand, Muang, Nonthaburi, Thailand; Division of Global Health Protection, Thailand MoPH-US CDC Collaboration, Ministry of Public Health, Nonthaburi, Thailand, Muang, Nonthaburi, Thailand; Mae Sot General Hospital, Mae Sot, Tak, Thailand, Mae Sot, Tak, Thailand; Nakhon Phanom Hospital, Nakhon Phanom, Thailand, Muang, Nakhon Phanom, Thailand; Division of Global Health Protection, Nakhon Phanom, Thailand, Muang, Nakhon Phanom, Thailand; Division of Global Health Protection, Tak, Thailand, Mae Sot, Tak, Thailand; Division of Global Health Protection, Nakhon Phanom, Thailand, Muang, Nakhon Phanom, Thailand; Division of Global Health Protection, Nakhon Phanom, Thailand, Muang, Nakhon Phanom, Thailand; Division of Global Health Protection, Tak, Thailand, Mae Sot, Tak, Thailand; Division of Global Health Protection, Thailand MoPH-US CDC Collaboration, Ministry of Public Health, Nonthaburi, Muang, Nonthaburi, Thailand; Department of Disease Control, Ministry of Public Health, Nonthaburi, Thailand, Muang, Nonthaburi, Thailand

## Abstract

**Background:**

Thailand’s national surveillance system to detect COVID-19 infection typically relies on a combination of fever, respiratory symptoms, and risk factors. We describe the frequency, epidemiologic characteristics, and risk factors for COVID-19 in patients without respiratory symptoms and the utility of saliva and nasal swabs (NS) to detect SARS-CoV-2.

**Methods:**

During June 2021-December 2022, a period of high COVID-19 incidence in Thailand, we enrolled patients aged >2 years presenting with a temperature ≥37.5 °C or a history of fever ≤14 days and no cough, dyspnea, or sputum production at 6 hospitals in Nakhon Phanom and Tak provinces, Thailand. Saliva, NS, and nasopharyngeal/oropharyngeal swabs (NP/OP) were collected to detect SARS-CoV-2 using real-time RT-PCR. Multivariable analysis was done to assess factors independently associated with COVID-19 infection. Cases were defined as persons with SARS-CoV-2-positive RT-PCR specimens.

**Results:**

Cases were detected in 358/1,648 (22%) persons. The median age among cases was 31 (IQR 21-45) years; 210 (59%) were female. Compared to non-cases, cases were more likely to present with malaise (52% vs. 40%, p< 0.001), hyposmia (10% vs. 1.4%, p< 0.001), dysgeusia (11% vs. 4.8%, p< 0.001), and muscle pain (53% vs. 47%, p=0.04); cases were less likely to have chills (41% vs. 50%, p=0.002), low appetite (29% vs. 41%, p< 0.001), nausea/vomiting (17% vs. 29%, p< 0.001) and abdominal pain (4.7% vs. 16%, p< 0.001). Cases were more likely to have been in contact with persons with COVID-19 infection (adjusted odds ratio [95% confidence interval]: 12 [8.1-16]), febrile household members (2.6 [1.8-3.8]), and persons with respiratory symptoms (2.2 [1.4-3.7]), have a history of travel (1.7 [1.0-2.7]), current alcohol consumption (1.7 [1.2-2.4]) and obesity (1.5 [1.1-1.9]). Among cases, SARS-CoV-2 was detected in 95% of saliva, 92% of NS, and 99% of NP/OP specimens; RT-PCR was positive in all 3 specimen types from 319 (89%) cases. Compared to NP/OP, the sensitivity of testing saliva and NS were 96% and 93%.

**Conclusion:**

SARS-CoV-2 was detected in more than one-fifth of febrile patients without respiratory symptoms. A broader national surveillance case definition would increase case detection. Saliva and NS demonstrated high sensitivity for SARS-CoV-2 detection.

**Disclosures:**

**All Authors**: No reported disclosures

